# Clinical, laboratory characteristics and growth outcomes of children with growing pains

**DOI:** 10.1038/s41598-022-19285-3

**Published:** 2022-09-01

**Authors:** Chung-Yuan Liao, Li-Chieh Wang, Jyh-Hong Lee, Kuan-Wen Wu, Yu-Tsan Lin, Yao-Hsu Yang, Bor-Luen Chiang, Hsin-Hui Yu

**Affiliations:** 1 Department of Pediatrics, National Taiwan University Children’s Hospital, Taipei, Taiwan; 2 Department of Pediatrics, Taitung Hospital, Ministry of Health and Welfare, Taitung, Taiwan; 3 Department of Orthopedic Surgery, National Taiwan University Hospital, Taipei, Taiwan; 4 Department of Medical Research, National Taiwan University Hospital, Taipei, Taiwan

**Keywords:** Paediatric research, Musculoskeletal system

## Abstract

Growing pains (GP), a common and benign pain syndrome of unknown etiology, is characterized by bilateral recurrent leg pain in childhood. There are no standardized diagnostic criteria for GP, and the diagnosis is often made by exclusion. To identify clinical and laboratory features, we included patients < 12 years with GP at National Taiwan University Children’s Hospital between April 2006 and April 2019 in a retrospective study. We also compared body weight and body height z-scores between diagnosis and up to 2 years post-diagnosis to determine if rapid growth was associated with GP. This cohort study included 268 patients with a mean age of 4.7 ± 2.2 years. The most common features of GP were bilateral leg pain, no limitation of activity, intermittent pain, normal physical examination, and being well physically. The average number of Walters' criteria fulfilled by the patients with GP was 6.7 ± 0.9. Elevated serum levels of alkaline phosphatase (ALP) and lactate dehydrogenase (LDH) were observed in 37.5% and 15.6% of patients, respectively. Symptomatic medications were used in 33% of patients. Our study indicates that ALP and LDH may be biomarkers associated with GP. There was no significant association between GP and rapid growth within 2 years of diagnosis.

## Introduction

Growing pains (GP) is a benign, non-inflammatory pain syndrome of childhood. There are no standardized and universally accepted diagnostic criteria or diagnostic tests for GP, and the diagnosis is often made by exclusion in clinical practice. The proposed combined diagnostic criteria for GP by Walters et al. described the key clinical features: (1) usually pain in both legs; (2) pain starts between ages 3 and 12 years; (3) pain typically occurs at the end of the day or during the night; (4) no limitation of activity nor limping; (5) typical distribution in the anterior thigh, calf, and posterior knee muscles; (6) intermittent pain with some pain-free days and nights; (7) normal physical examination, no evidence of orthopedic disorders, trauma, or infections; (8) unremarkable results of laboratory tests (such as erythrocyte sedimentation rate), radiograph and bone scan; (9) pain for at least 3 months; and (10) no associated lack of well-being^1^.

GP is the most common cause of musculoskeletal pain in early childhood. Previous studies showed a wide range of estimated prevalence from 2.6 to 49.4% due to different definitions of GP, poor population sampling, and disparate age ranges^2–6^. GP is prevalent in children aged 4–6 years^4^. Although familial cases are common, the etiology and pathophysiology of GP are still unclear. Laboratory tests usually yield normal results^7^. The prognosis is good as GP is self-limiting, and most of the symptoms resolve by adolescence^8^. The current study analyzed the clinical features, laboratory findings, and growth outcomes of Taiwanese children with GP.

## Methods

This study was conducted retrospectively at National Taiwan University Children’s Hospital, a large medical center. Patients aged less than 12 years who presented with intermittent leg pain from April 2006 to April 2019 were enrolled based on clinical diagnosis of GP^1^. The exclusion criteria were symptoms and signs of arthritis (erythema, swelling, warmth, and/or tenderness in the joints), limping gait or limitation of activity, abnormal radiograph findings, abnormal inflammatory markers (such as C-reactive protein or erythrocyte sedimentation rate), or any systemic disease. Clinical features, especially the fulfillment of Walters’ diagnostic criteria, laboratory data, and medication records were analyzed. This study was approved by National Taiwan University’s Hospital Research Ethics Committee (IRB approval number: 201812007RIND). Informed consent was waived by National Taiwan University’s Hospital Research Ethics Committee because of the retrospective nature of the study and the analysis used anonymous clinical data. All experiments were performed in accordance with relevant guidelines and regulations.

Body weight (BW), body height (BH), and body mass index (BMI) were collected from every patient’s medical record at every visit. Each value was transformed to the percentile and z-score based on the growth curves of the WHO and Taiwanese Child Growth Standards^9–11^. To test the association between GP and BH or BW, we compared the baseline BH or BW with BH or BW at 1 year before diagnosis, and 6 months, 1 year, and 2 years after diagnosis of GP, respectively.

Data analysis was conducted using SPSS Statistics for Windows (version 22.0). Z-scores for BH, BW, and BMI were calculated using a macro for SPSS obtained from the WHO website^12^. Independent samples t-test was used to compare continuous variables. Fisher’s exact test was used for categorical variables. Paired samples t-test was used to compare percentiles and z-scores assessed at two different times. Z-score is a score indicating how many standard deviations an observation is from a population median, adjusted for age and sex. The figures were generated by GraphPad Prism (version 7.0). A *P* value < 0.05 was considered significant.

## Results

**Figure 1:**
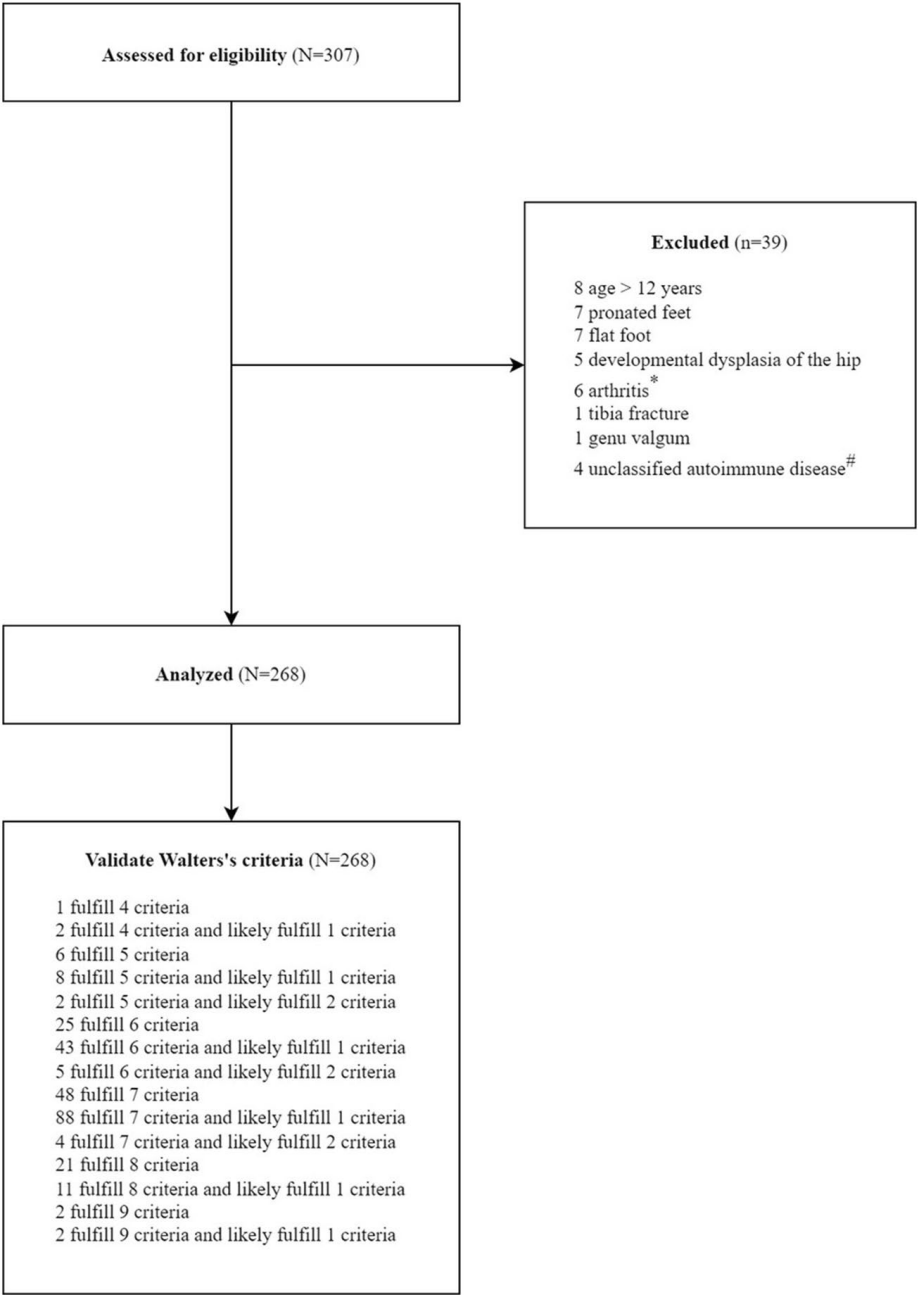
Study flow chart.

**Figure 2:**
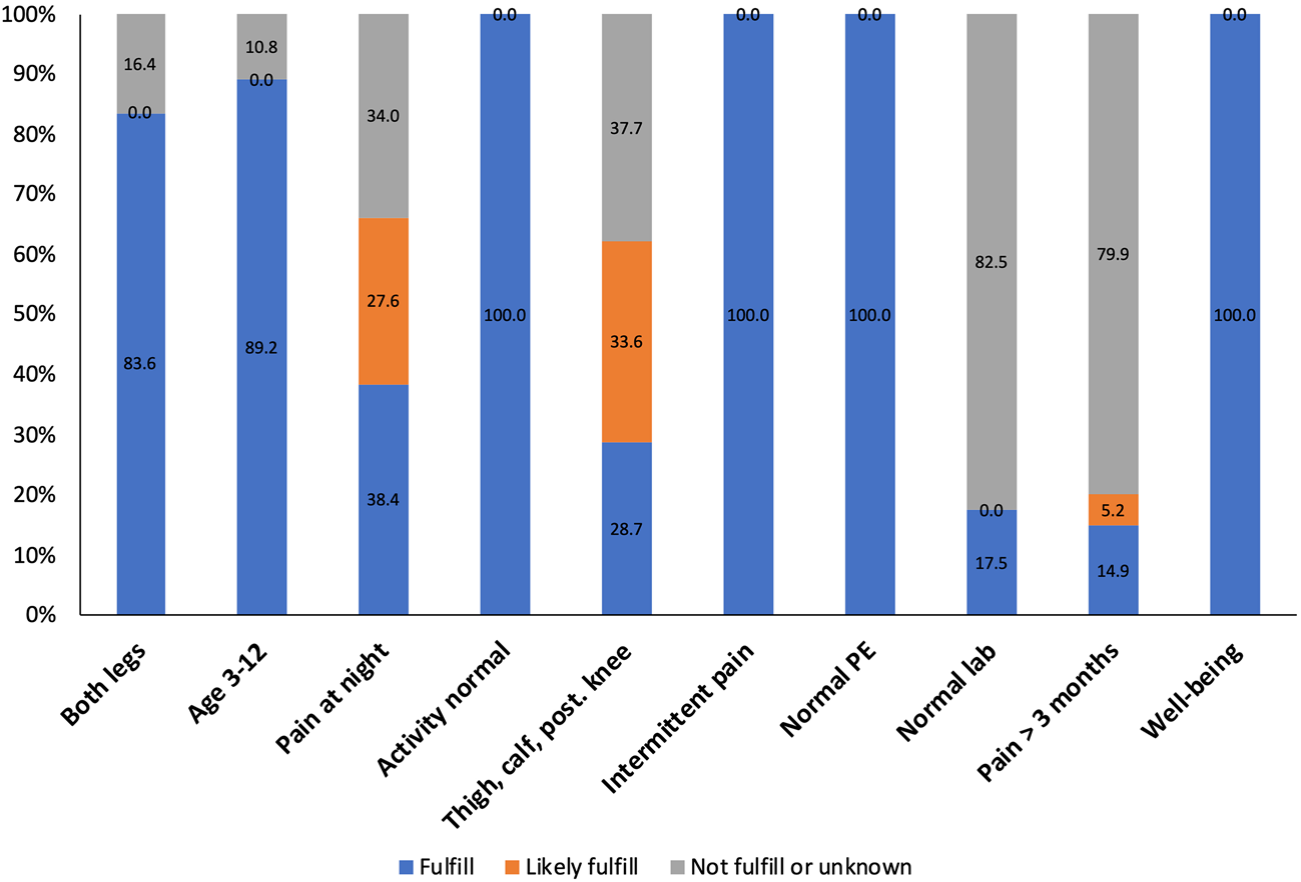
Percentage of fulfillment, likely fulfillment, and no fulfillment of ten Walters's criteria of growing pains.

This study identified 307 patients with intermittent leg pain. Eight patients older than 12 years were first excluded (Fig. 1). After excluding 31 patients with other musculoskeletal diagnoses, 268 patients were included and analyzed. The excluded patients consisted of the following final diagnoses: 7 with pronated feet, 7 with flat feet, 5 with developmental dysplasia of the hip, 1 with tibia fracture, 1 with genu valgum, and 6 with arthritis (3 with juvenile idiopathic arthritis (JIA), 1 with IgA vasculitis), and 4 with unclassified autoimmune disease (Fig. 1, Supplementary Fig. 1). In our study population, every patient fulfilled more than four Walters' criteria for GP. On average, the patients fulfilled 6.72 ± 0.85 criteria and likely fulfilled 0.66 ± 0.56 criteria. The four most common features were no limitation of activity and no limping, intermittent pain with some pain-free days and nights, normal physical examination, and no lack of well-being (100%) (Figs. 1, 2).

The mean age of patients with GP was 4.65 ± 2.17 years (mean ± standard deviation) with slight male predominance (54%). For clinical presentation, 226 patients (84.3%) had bilateral leg pain, and all had pain in the lower extremities. Symptomatic treatments were used in 88 patients (33%), including 34 patients (12.7%) treated with only oral acetaminophen or non-steroidal anti-inflammatory drugs, 50 patients (18.7%) treated with only topical analgesics, and three patients (1.1%) received both treatments (Table 1).

**Table 1:** Demographic and clinical characteristics of children with growing pains.

	N (%) or values
Total number of patients	268 (100)
Male	146 (54.5)
Age, years	4.65 ± 2.17 (1.17–12.39)
Body weight, kg	18.52 ± 6.95 (9.5–66.0)
Body height, cm	105.47 ± 14.91.08 (80.0–153.0)
BMI, kg/m^2^	16.04 ± 3.11 (13.2–28.19)
**Clinical presentation**	
Bilateral pain	226 (84.3)
Onset of pain	
Morning	7/163 (4.3)
Afternoon	5/163 (3.1)
Night	151/163 (92.6)
Location of pain	
Upper limbs	12 (4.5)
Lower limbs	268 (100)
Limitation of activity	0 (0)
**Examination**	
Plain film	65 (24.3)
Laboratory tests	72 (26.9)
**Management**	
Observation only	181 (67.5)
Oral medications only	34 (12.7)
Topical medications only	50 (18.7)
Both oral and topical medications	3 (1.1)
Follow-up	70 (26.1)

*BMI* body mass index.

*The values are expressed as mean ± standard deviation (range).

For diagnostic tests, plain radiographs were performed in 24.3% of patients. Laboratory tests were also performed in 26.9% of patients. Elevated alkaline phosphatase (ALP) levels were found in 6 out of 16 patients (37.5%) using age-adjusted normal ranges of ALP, and none had elevation above 1.5 times the upper limit of the normal range. Elevated serum levels of lactate dehydrogenase (LDH) were observed in 15.6% of patients. Patients with elevated serum ALP levels had significantly higher mean levels of LDH (549.40 ± 116.64 vs. 233.33 ± 34.86 U/L, *P* = 0.003) (Table 2; Supplementary Table 2). The mild elevation of white blood cell count and aspartate aminotransferase (AST) levels decreased to normal levels after 3–4 months of follow-up. There were three patients (6.1%) with abnormal antinuclear antibodies titers, one of which was 1:1280, and the titers became 1:80 after a 4-month follow-up.

**Table 2:** Laboratory features in patients with growing pains.

Parameter	Number, n	Mean value*	Elevated, n (%)
WBC (k/μL)	70	8.31 ± 2.33	3 (4.3)
Hemoglobin (g/dL)	69	12.89 ± .0.91	7 (10.1)
Platelet (k/μL)	69	337.90 ± 83.40	6 (8.7)
CRP (mg/dL)	53	0.10 ± 0.23	0 (0)
ESR (mm/h)	45	10.06 ± 7.58	0 (0)
LDH (U/L)	45	397.53 ± 208.45	7 (15.6)
AST (U/L)	25	32.88 ± 9.18	4 (16.0)
ALT (U/L)	21	13.24 ± 4.79	0 (0)
CK (U/L)	19	107.89 ± 29.09	0 (0)
ALP (U/L)	16	308.31 ± 159.76	6 (37.5)
C3 (mg/dL)	49	113.65 ± 19.61	0 (0)
C4 (mg/dL)	49	20.88 ± 7.76	0 (0)
ANA	49		3 (6.1)
RF (IU/mL)	24	All negative	
HLA-B27	5	All negative	

*WBC* white blood count, *CRP* C-reactive protein, *ESR* erythrocyte sedimentation rate, *LDH* lactate dehydrogenase, *AST* aspartate aminotransferase, *ALT* alanine aminotransferase, *CK* creatine kinase, *ALP *alkaline phosphatase, *C3* Complement 3, *C4* Complement 4, *ANA* antinuclear antibodies, *RF* rheumatoid factor, *HLA-B27* human leukocyte antigen B27. The values are expressed as mean ± standard deviation.

**Figure 3:**
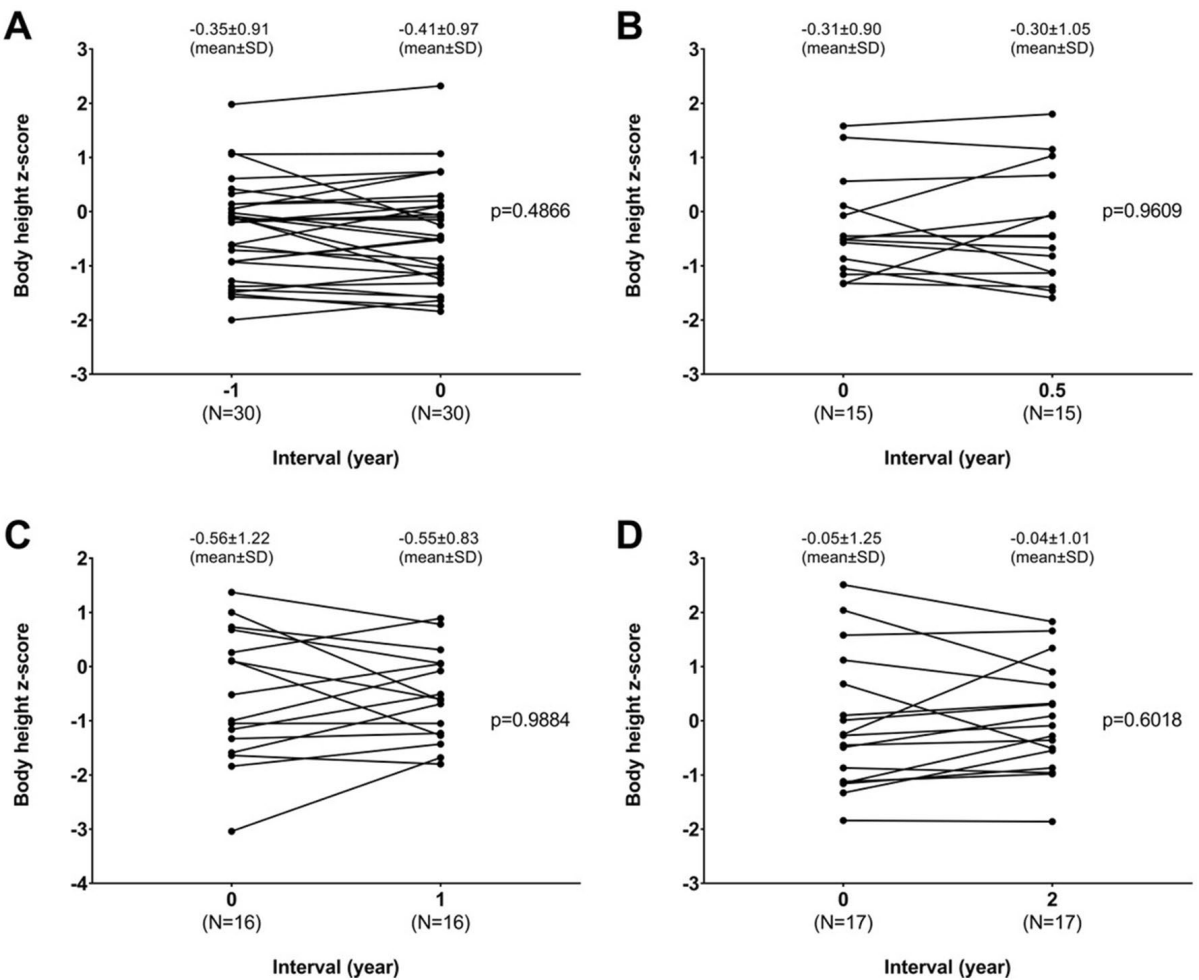
Changes in body height z-scores at different time intervals from diagnosis of growing pains (GP). The time interval is defined as the time elapsed between diagnosis of GP and record of body height at another visit, with 0 being the point at which GP was diagnosed. Paired samples t-test showed no significant differences in body height z-scores between the time of diagnosis and prior or subsequent time points: (a) 1 year before the diagnosis of GP, (**b**) 6 months after diagnosis, (**c**) 1 year after diagnosis, and (**d**) 2 years after diagnosis.

All patients recovered without significant long-term disabilities or co-morbidities. The distribution of BH z-scores at different time intervals from diagnosis is shown in Fig. 3. Compared to BH z-scores at the time of diagnosis of GP (− 0.41 ± 0.97, interval 0), there were no significant differences in z-scores 1 year before (− 0.35 ± 0.91), 6 months (− 0.30 ± 1.05), 1 year (− 0.55 ± 0.83), or 2 years (− 0.04 ± 1.01) after the diagnosis of GP, respectively (Fig. 3, Supplementary Fig. 2). The BW and BMI z-scores did not have significant differences between these time points and the time of diagnosis of GP (Supplementary Table 3).

## Discussion

We demonstrated the clinical features of GP in a large cohort of Taiwanese children with a mean age at diagnosis of 4.65 years. In our study, every patient fulfilled at least four Walters' diagnostic criteria for GP. Because GP usually affects younger children, patients older than 12 years were excluded from our study. In adolescents with similar symptoms, a diagnosis such as juvenile fibromyalgia, spinal problems, and stress fractures should be considered.

Researchers observed an overlap between GP and restless legs syndrome (RLS) (Willis–Ekbom disease) in children. RLS is a neurological sensorimotor disease that profoundly disturbs sleep and quality of life. However, the association between GP and RLS ranges from no association to a substantial overlap. GP is experienced in 27–80.6% of children with RLS^1^. Discriminating pediatric RLS from GP is assisted by the criteria of the International RLS Study Group, which cites “the urge to move” as characteristic of RLS, and not reported in GP^13^. While RLS continues across the lifespan and is associated with mood, sleep, blood pressure, learning, and behavioral problems, GP is confined to childhood, self-limiting and benign in nature^14^. None of our patients fulfilled the mandatory diagnostic criteria for childhood RLS (Supplementary Table 1). No patients developed fibromyalgia or sleep disorder, either.

There are several other proposed hypotheses for GP, including anatomical factors such as flat feet, over-pronated feet, and joint hypermobility^15–19^, lower pain threshold^20,21^, lower skeletal vascular perfusion^15,22^, reduced bone strength^23,24^, and psychological factors^25–27^. Various factors, individually or in association, might be responsible for the onset of GP. In our study, we excluded 31 patients with other musculoskeletal diagnoses or autoimmune diseases. Normal levels of antinuclear antibodies, rheumatoid factor, CRP, and ESR could exclude autoimmune diseases, especially JIA. Careful evaluation by orthopedic surgeons for anatomical abnormalities in children with aching legs is important^28^. In a study by Lee et al., over-pronated feet account for 75% of pediatric patients with GP, and pain episodes were significantly reduced using foot orthoses^18^.

We found that serum levels of ALP and LDH were elevated in 37.5% and 15.6% of patients with GP, respectively. Serum ALP level was recognized as an indicator of osteoblastic activity and a valid marker of active bone formation. ALP level is higher in growing children than fully grown adults, and the highest ALP level is detected during infancy and puberty^29^. Our patients with elevated ALP levels have similar distributions in age, sex, BH, BW, and BMI, compared with patients with normal ALP levels. Patients with GP did not have rapid growth of body height and weight within 2 years of onset of symptoms in our study. In our study, patients with elevated ALP levels had significantly higher levels of LDH at diagnosis. Patients with elevated ALP and LDH levels could have mild inflammation that may cause musculoskeletal pain.

ALP expression is regulated by 1,25 (OH)_2_-vitamin D, retinoic acid, and parathyroid hormone^30^. Hypovitaminosis D reduces calcium absorption and serum calcium levels, triggering secondary hyperparathyroidism, which subsequently increases bone resorption and serum ALP level^31^. In an observational study by Qamar et al., elevated ALP, vitamin D insufficiency, and vitamin D deficiency were found in 38%, 22%, and 72% of 100 children with GP^32^. Most patients (97.4%) with GP and elevated ALP had vitamin D deficiency^32^. However, the association between serum ALP levels and GP was insignificant in previous studies^7,33,34^. In GP, whether there is a causal relationship with hypovitaminosis D or whether hypovitaminosis D is a risk factor for severe pain through peripheral and central mechanisms is unclear. A high prevalence (57–94%) of hypovitaminosis D was found among the children with GP compared to the prevalence in the general population in children^35–38^. Oral vitamin D supplementation has been shown to be effective in reducing pain severity in children with GP, although there were no control groups for comparison in these studies^35–37^.

To our knowledge, this is the first study that validated Walters’ diagnostic criteria for GP in a large pediatric cohort. Study strengths included high diagnostic accuracy by the exclusion of musculoskeletal and autoimmune diseases that were diagnosed by specialists. Our study did have weaknesses, including missing information regarding laboratory data, BH, and BW during follow-up. It also lacked a group of healthy children to compare laboratory parameters, especially the levels of ALP and LDH. The current study compared laboratory parameters with an age-adjusted normal range in our hospital. The major limitation of our study was that we did not check serum vitamin D3 and parathyroid hormone (PTH) levels at disease diagnosis. Therefore, we could not clarify the association between hypovitaminosis D and GP in our cohort due to the retrospective study design. We speculated that vitamin D deficiency may be a risk factor for GP. However, the elevation of ALP or LDH might be associated with stimulated bone growth or mild inflammation in certain patients with GP. Further large-scale prospective case–control studies to investigate the association between vitamin D, PTH, ALP, LDH levels, and the severity of GP are warranted.

In conclusion, GP is a common and self-limited leg pain syndrome in children. It was not associated with RLS syndrome in our cohort. ALP and LDH may be potential biomarkers associated with the disease, and mild inflammation may cause musculoskeletal pain. There was no significant association found between GP and rapid growth or higher BMI within 2 years of diagnosis.

## Supplementary Information

Supplementary Information.

## Data Availability

The datasets generated during and/or analyzed during the current study are available from the corresponding author on reasonable request.
