# Supplementary material for: Clinical, laboratory characteristics and growth outcomes of children with growing pains
Source: Sci Rep. 2022 Sep 1;12:14835. doi: 10.1038/s41598-022-19285-3 (PMC9436948; doi:10.1038/s41598-022-19285-3)

Supplementary Table 1. Fulfillment of the childhood restless legs syndrome 2012 revised criteria

**Diagnostic criteria for childhood restless legs syndrome***	Fulfill, n (%)	Likely fulfill, n (%)	Not fulfill or unknown, n (%)
An urge to move the legs, usually accompanied or caused by uncomfortable and unpleasant sensations in the legs	0 (0)	0 (0)	268 (100)
The urge to move or unpleasant sensations begin or worsen during periods of rest or inactivity such as lying down or sitting	0 (0)	151 (56.3)	117 (43.7)
The urge to move or unpleasant sensations are partially or totally relieved by movement, such as walking or stretching, at least as long as the activity continues	1 (0.4)	44 (16.4)	223 (83.2)
The urge to move or unpleasant sensations are worse in the evening or night than during the day, or only occur during the evening or night	151 (56.3)	0 (0)	117 (43.7)

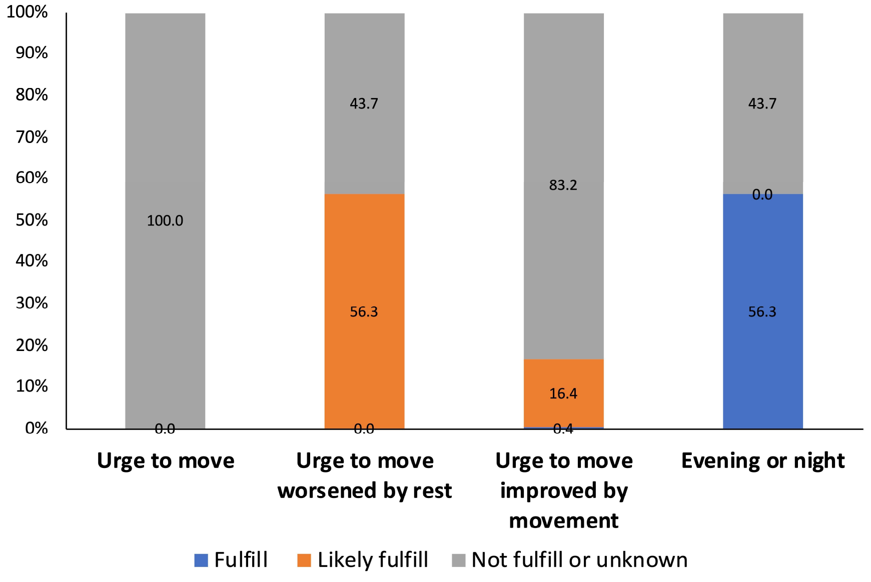

Supplementary Table 2. Comparison of clinical and laboratory features between patients with normal and elevated alkaline phosphatase levels

	N (%) or values*#		*p* value
	Normal ALP (N=10)	Elevated ALP (N=6)	
Male	6 (60)	1 (17)	0.145
Age (years)	5.60 ± 3.169	5.67 ± 2.422	0.965
Clinical presentation			
Bilateral pain	9 (90)	5 (83.3)	
Onset of pain			
Morning	0 (0)	0 (0)	
Afternoon	1 (10)	0 (0)	
Night	9 (90)	6 (100)	
Symptomatic treatment	3 (30)	0 (0)	
Follow-up	6 (60)	4 (66.7)	
Laboratory data			
WBC (k/uL)	8.86 ± 3.05	7.06 ± 1.82	0.219
Hemoglobin (g/dL)	12.91 ± 0.90	13.60 ± 1.05	0.197
Platelet (k/uL)	372.00 ± 107.52	348.00 ± 97.05	0.668
CRP (mg/dL)	0.04 ± 0.03	0.05 ± 0.04	0.594
ESR (mm/hr)	9.33 ± 5.13	12.00 ± 2.83	0.415
AST (U/L)	27.00 ± 5.35	32.50 ± 0.71	0.210
ALT (U/L)	12.67 ± 3.39	14.00 ± 1.41	0.471
CK (U/L)	80.00	135.00	
ALP (U/L)	197.60 ± 32.77	492.83 ± 96.27	
C3 (mg/dL)	113.73 ± 19.26	126.20 ± 15.50	0.274
C4 (mg/dL)	19.85 ± 5.93	26.96 ± 11.03	0.204
LDH (U/L)	233.33 ± 34.86	549.40 ± 116.64	**0.003**

N of elevated LDH	0 (0)	3 (50)

WBC white blood count, CRP C-reactive protein, ESR erythrocyte sedimentation rate, AST aspartate aminotransferase, ALT alanine aminotransferase, CK creatine kinase, ALP alkaline phosphatase, C3 Complement 3, C4 Complement 4, LDH lactate dehydrogenase

* The values are expressed as mean ± standard deviation

The total "N" for this table was 16

Supplementary Table 3. Body size parameters at different time intervals from diagnosis of growing pains*

	Time interval (year)[#]				
	-1 (n=30)	0 (n=116)	0.5 (n=15)	1 (n=17)	2 (n=17)
BH					
in cm	100.41± 10.99	105.01 ± 12.79	105.40 ± 11.70	107.01 ± 7.49	118.82 ± 13.03
in percentile	38.54 ± 26.38	42.67 ± 31.02	40.70 ± 31.80	36.93 ± 29.06	53.05 ± 29.74
z-score	-0.35 ± 0.91	-0.22 ± 1.18	-0.30 ± 1.05	-0.42 ± 0.96	0.04 ± 1.01
BW					
in kg	15.69 ± 3.05	17.25 ± 4.66	16.92 ± 3.84	17.82 ± 2.33	22.81 ± 7.07
in percentile	46.23 ± 27.97	45.13 ± 28.84	38.40 ± 21.40	45.14 ± 31.69	52.14 ± 32.72
z-score	-0.19 ± 1.09	-0.16 ± 1.05	-0.33 ± 0.61	-0.17 ± 1.07	0.03 ± 1.10
BMI					
in kg/m^2	15.68 ± 1.86	15.50 ± 1.89	15.03 ± 0.75	15.56 ± 1.50	15.80 ± 2.34
in percentile	55.02 ± 30.35	47.33 ± 31.48	43.55 ± 21.47	52.73 ± 27.51	50.05 ± 36.28
z-score	0.10 ± 1.38	-0.05 ± 1.34	-0.22 ± 0.68	0.13 ± 1.08	-0.02 ± 1.37

BH body height, BW body weight, BMI body mass index

[*] The values are expressed as mean ± standard deviation

[#] The time interval is defined as the time (year) elapsed between diagnosis of growing pains and record of body height, weight and BMI at another visit, with 0 being the point at which growing pains was diagnosed. Patients with negative elapsed time had records of body height, weight and BMI before they were diagnosed as having growing pains. None of the comparison between the time of diagnosis and other time points had statistically significant difference.

Supplementary Figure 1. Images of excluded cases. (a).Radiograph of hip showing abnormal angles between the femoral head and the acetabulum bilaterally, which indicates bilateral dysplasia of the hip in a 6-year-old boy with bilateral hip and leg pain. (b) Radiograph of the left leg showing tibia fracture in a 4-year-old boy with left leg pain.

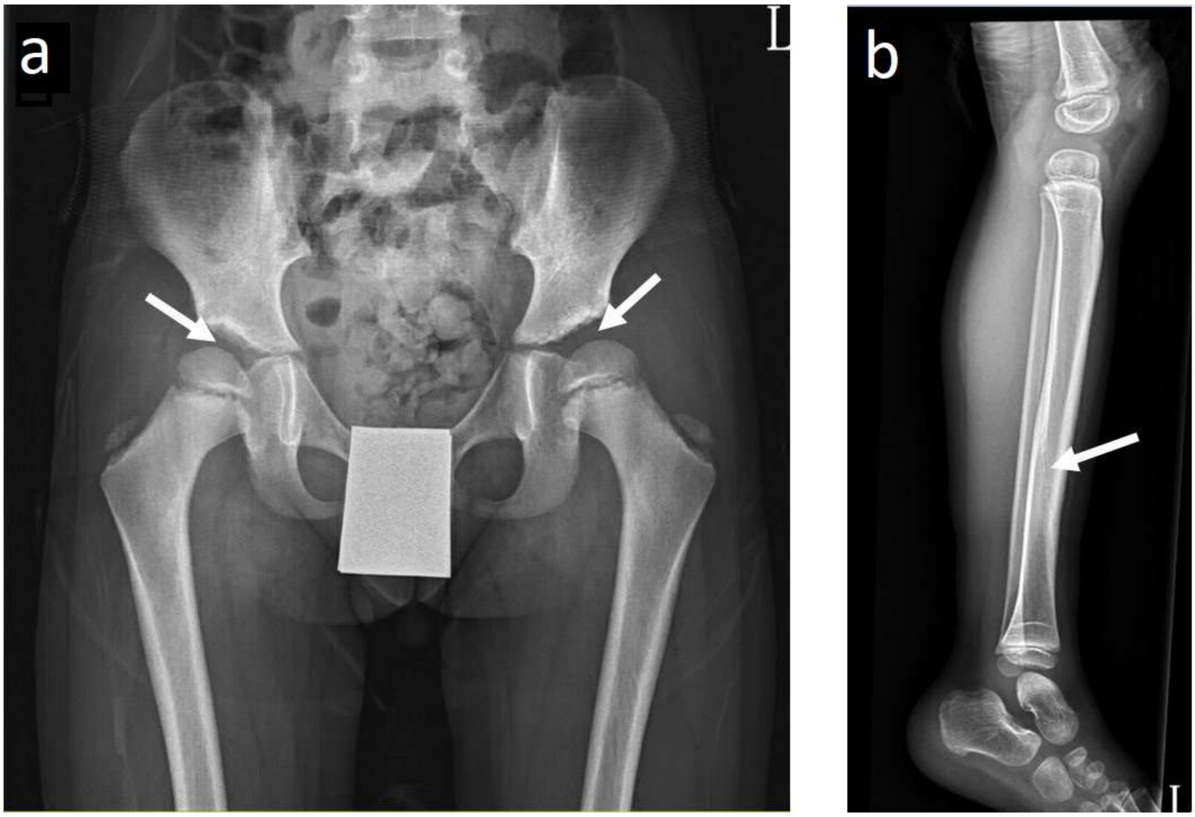

Supplementary Figure 2. **Box and whisker plots of body height z-scores at different time intervals from diagnosis.** The time interval is defined as the time elapsed between diagnosis of growing pains and record of body height at another visit, with 0 being the point at which growing pains was diagnosed. Patients with negative elapsed time had records before they were diagnosed with growing pains.

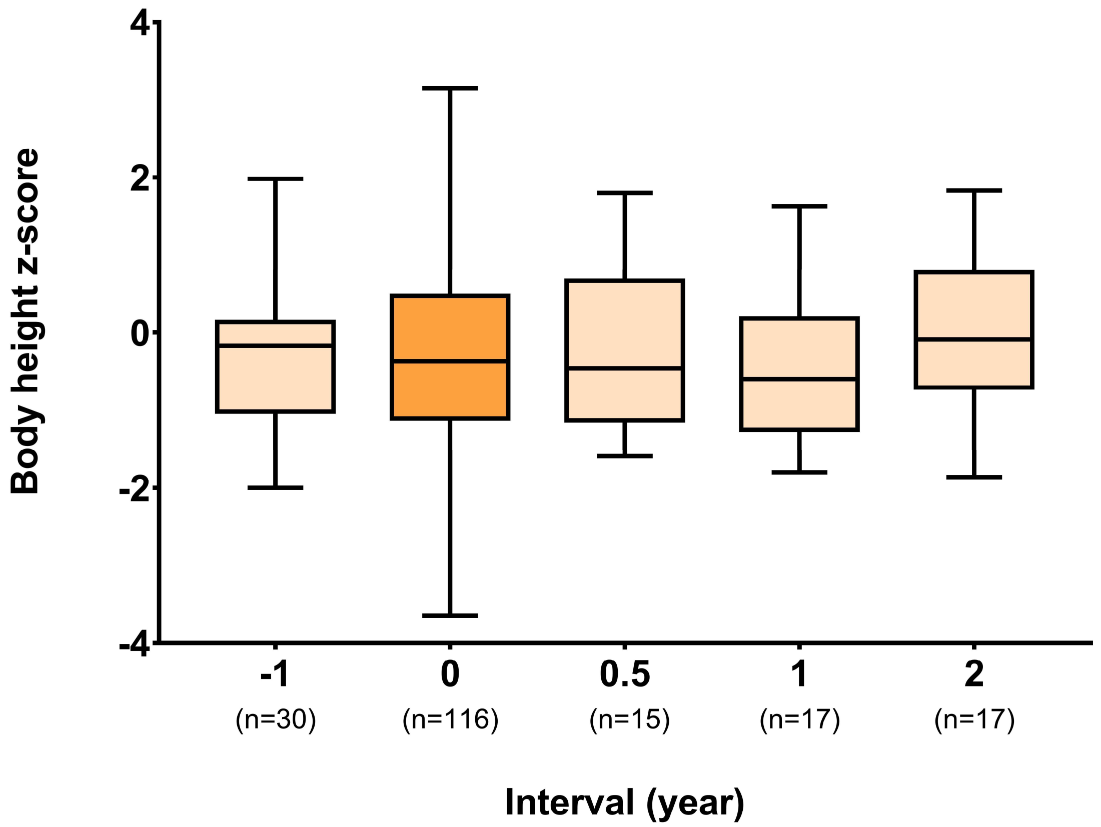

Supplement: Supplementary file 1 — Supplementary Information. [file 41598_2022_19285_MOESM1_ESM.pdf]
